# NanoSPC: a scalable, portable, cloud compatible viral nanopore metagenomic data processing pipeline

**DOI:** 10.1093/nar/gkaa413

**Published:** 2020-05-22

**Authors:** Yifei Xu, Fan Yang-Turner, Denis Volk, Derrick Crook

**Affiliations:** Nuffield Department of Medicine, University of Oxford, John Radcliffe Hospital, Oxford OX3 9DU, UK; NIHR Oxford Biomedical Research Centre, University of Oxford, UK; Nuffield Department of Medicine, University of Oxford, John Radcliffe Hospital, Oxford OX3 9DU, UK; NIHR Oxford Biomedical Research Centre, University of Oxford, UK; Nuffield Department of Medicine, University of Oxford, John Radcliffe Hospital, Oxford OX3 9DU, UK; NIHR Oxford Biomedical Research Centre, University of Oxford, UK; Nuffield Department of Medicine, University of Oxford, John Radcliffe Hospital, Oxford OX3 9DU, UK; NIHR Oxford Biomedical Research Centre, University of Oxford, UK

## Abstract

Metagenomic sequencing combined with Oxford Nanopore Technology has the potential to become a point-of-care test for infectious disease in public health and clinical settings, providing rapid diagnosis of infection, guiding individual patient management and treatment strategies, and informing infection prevention and control practices. However, publicly available, streamlined, and reproducible pipelines for analyzing Nanopore metagenomic sequencing data are still lacking. Here we introduce NanoSPC, a scalable, portable and cloud compatible pipeline for analyzing Nanopore sequencing data. NanoSPC can identify potentially pathogenic viruses and bacteria simultaneously to provide comprehensive characterization of individual samples. The pipeline can also detect single nucleotide variants and assemble high quality complete consensus genome sequences, permitting high-resolution inference of transmission. We implement NanoSPC using Nextflow manager within Docker images to allow reproducibility and portability of the analysis. Moreover, we deploy NanoSPC to our scalable pathogen pipeline platform, enabling elastic computing for high throughput Nanopore data on HPC cluster as well as multiple cloud platforms, such as Google Cloud, Amazon Elastic Computing Cloud, Microsoft Azure and OpenStack. Users could either access our web interface (https://nanospc.mmmoxford.uk) to run cloud-based analysis, monitor process, and visualize results, as well as download Docker images and run command line to analyse data locally.

## INTRODUCTION

Oxford Nanopore Technology (ONT) is a third generation sequencing technology with the advantage of generating real-time, long read data with highly portable devices. Nanopore sequencing has been successfully applied in a broad range of research areas, including human genetics, cancer, microbiology, plant, and infectious diseases (1–5). On-site sequencing with ONT has enabled real-time surveillance and tracking of Ebola, Zika and Lassa epidemics (6–8).

Metagenomic sequencing has the capacity to detect all potential pathogens from individual clinical samples, and provide genomic information for comprehensive characterization of the pathogens, microbiome analyses, and investigation of epidemiology and transmission (9–11). Metagenomic sequencing with ONT has the potential to become a point-of-care test for infectious diseases in clinical and public health settings, providing rapid diagnosis of infection, guide individual patient management and treatment strategies, and informing infection prevention and control practices (12–14). Nanopore metagenomic sequencing of the novel coronavirus disease 2019 (COVID-19) that causes the ongoing pandemic in the world has provided critical and timely evidence for human-to-human transmission of this virus (15).

Nanopore sequencing data is characterized by high error rates (approximately 10% with state of art chemistry and basecalling algorithms) compared to next generation sequencing (approximately 0.1%) (16). A variety of bioinformatic tools have been developed to overcome such high error rates and improve data quality for each step associated with the analysis, including basecalling, reads mapping, variants calling, and genome assembly (17–21). However, publicly available streamlined and reproducible pipelines for analyzing Nanopore metagenomic sequencing data are still lacking, which consequently could impede its application, especially for users with minimum bioinformatics knowledge.

Here, we introduce NanoSPC, a scalable, portable and cloud compatible pipeline for analyzing Nanopore sequencing data. NanoSPC can identify potentially pathogenic viruses and bacteria simultaneously to provide comprehensive characterization of individual samples. The pipeline can also detect single nucleotide variants and assemble high quality complete consensus genome sequences, which permits inference of transmission with high resolution. We implement NanoSPC using Nextflow pipeline manager within Docker images to allow reproducibility and portability of the analysis. We deploy NanoSPC to our scalable pathogen pipeline platform, enabling elastic computing for high throughput data via HPC cluster as well as multiple commercial cloud platforms.

## METHODS AND FUNCTIONALITIES

### Data input and quality control

**Figure 1.**
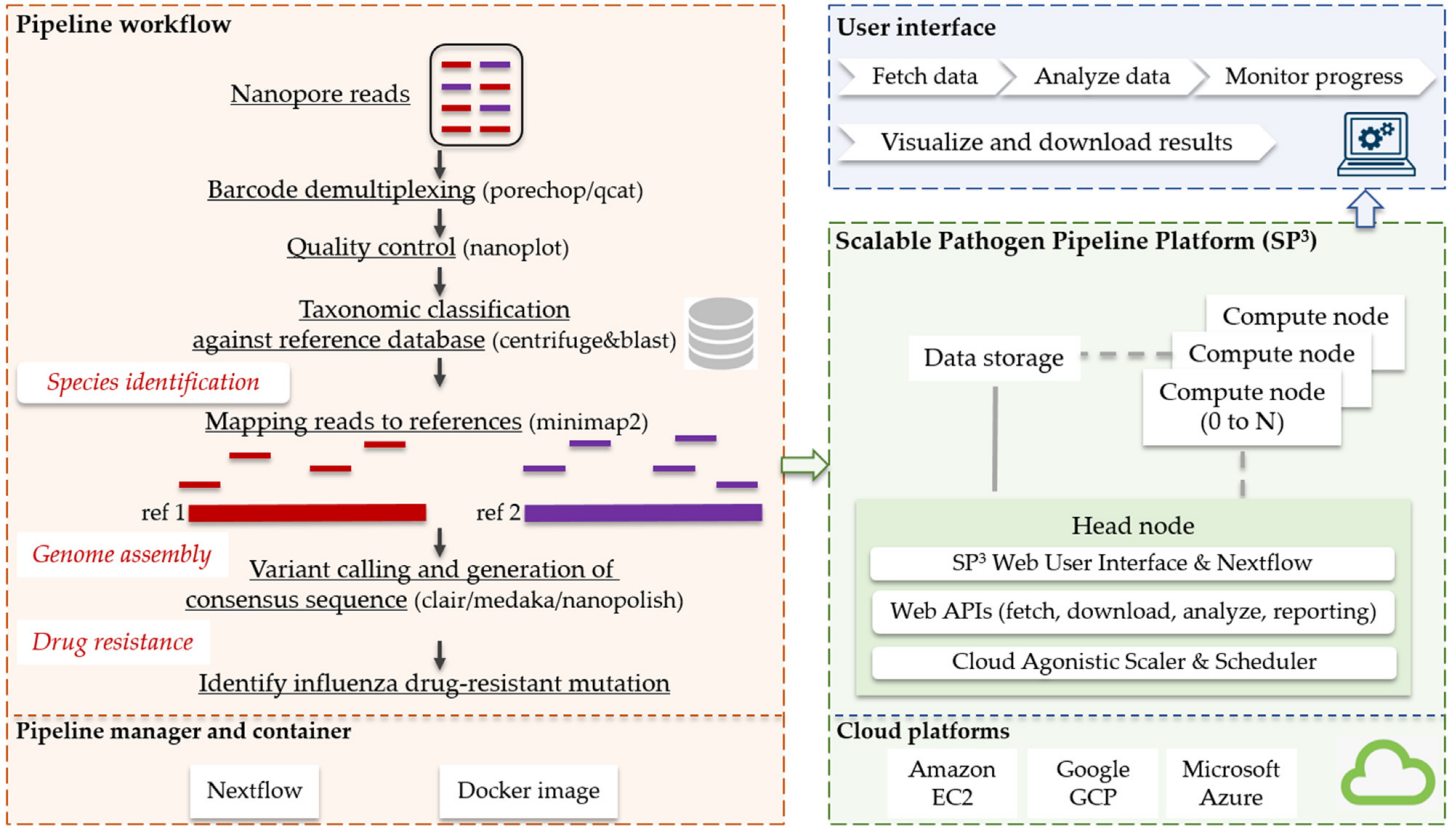
A schematic overview of NanoSPC. NanoSPC is a scalable, portable, and cloud compatible pipeline that can analyse the Nanopore metagenomic sequencing data. NanoSPC can identify potentially pathogenic viruses and bacteria simultaneously to provide comprehensive characterization of individual samples. The pipeline can also detect single nucleotide variants and assemble high quality complete consensus genome sequences. NanoSPC uses Nextflow pipeline manager and packs all the software dependencies within Docker images (red). NanoSPC can be deployed into the scalable pathogen pipeline platform, enabling elastic computing for high throughput Nanopore data on multiple cloud platforms (green). NanoSPC can be accessed via a web interface to run cloud-based analysis as well as Docker images to analyze data locally (blue).

NanoSPC takes Nanopore sequencing reads of the fastq format and, optionally, raw signal data of the fast5 format as input (Figure 1). We have tested the pipeline with fastq reads produced by the R9.4 and R9.4.1 version flow cells, native and rapid barcoding kits. The acceptable multi-read fast5 format should contain data pertaining to multiple reads in each fast5 file. While NanoSPC is designed to analyze Nanopore sequencing data, some of its modules could be used to analyze sequencing data from other platforms, such as PacBio or Illumina. The pipeline investigates the quality of the sequencing reads using NanoPlot (22). A comprehensive statistical summary is produced to report the overall data quality, including number of reads, total nucleotide bases, mean and median read length, and quality scores. In addition, a variety of informative graphs are generated to display multiple aspects of the data, such as cumulative yield plot showing efficiency of the flow cell against time, heat map of the physical layout of the flow cell comparing the efficiency of each channel, and violin plots showing base call quality against time.

### Identification of species

Metagenomic sequencing data are generally associated with a high level background. In order to identify potentially pathogenic viruses and bacteria with high sensitivity and specificity, we implement a method that combines taxonomic classification, reference based mapping, and filtering.

NanoSPC first applies Centrifuge v1.0.3 (23) to classify sequencing reads to a taxonomic identifier in the centrifuge reference database (p_compressed+h+v) that comprises 20 174 complete bacterial, archaeal, viral, and human genomes corresponding to 11 539 taxonomic IDs. One primary assignment with a score >150 is reported for individual reads. Based on the centrifuge report, the pipeline selects a draft reference genome for each viral species, and maps sequencing reads to the draft reference using Minimap2 (24). In order to optimize the reference sequence for viral species, a preliminary consensus sequence for each viral species is generated using a simple majority voting method that selects the most abundant base at each genomic position. The preliminary consensus sequences are then BLASTed against a customized viral reference database to determine more optimal reference sequences. Finally, we map the reads to these references using Minimap2 for a second time. The customized viral reference database comprises of >86 000 complete genomes of viral pathogens downloaded from NIAID Virus Pathogen Database and Analysis Resource (ViPR) (25) in March 2020. We aim to update this viral reference database every four months or when significant novel pathogen species are discovered.

To distinguish species present in the data and artifacts, the pipeline implements the following filtering of the mapping results: (I) retain mapped reads with a mapping quality >50; (II) retain reads that have >80% of the bases mapped to the reference sequence (i.e. if the length of a read is 1000 bp, >800 bp are required to be mapped). Viral species with ≥2 mapped reads or one mapped read longer than 400 bp are reported.

### Genome assembly and variant calling

Whole genome sequencing can provide high-resolution investigation of transmission and characterization of the spatiotemporal spread of outbreaks. While low accuracy on the sequencing read level (∼90%) is a major limitation of nanopore sequencing, genome assembly can generate high-accuracy consensus sequence with adequate sequencing depth. The full genome consensus sequences generated by NanoSPC have been used to delineate nosocomial transmission of influenza A virus and human metapneumovirus, contributing to improvement of infection prevention and control practices (26).

NanoSPC enables three variant calling modes, namely nanopolish, medaka and clair. With the nanopolish mode, NanoSPC takes both sequencing reads of the fastq format and raw signal data of the fast5 format as inputs, and applies Nanopolish (19) to detect single nucleotide variants. The pipeline considers candidate variants from the aligned reads when the variant frequency is >10% and the mapping depth is >10. To distinguish true variants and artifacts, NanoSPC keeps variants that the number of signal reads used to call the variant are >10 and the fraction of signal reads that support the variant are >75%. Finally, reads are mapped against the consensus sequence and only positions that are supported by >70% of mapped reads are kept.

While the nanopolish mode can generate consensus sequence with high accuracy, it is computationally intensive and time consuming, due to the large size of fast5 signal data. Therefore, signal based variant calling methods present a challenge to data storage, transfer, and analysis. Fortunately, with the Medaka and clair mode, NanoSPC applies Medaka (ONT) and clair (27) to detect single nucleotide variants. The advantages of these two modes is that they only take fastq format sequencing reads, and can be competitive with the nanopolish mode and being much faster.

The parameter values that we describe in the methods and functionalities section are selected based on publications (6,28) and our recent studies (26,29).

### Influenza drug resistance analysis

If influenza viruses are identified from the sequencing data, the pipeline determines the type of influenza virus (A or B) and the HA and NA subtype of influenza A virus based on the reference sequences. The pipeline analyzes assembled consensus sequences of Neuraminidase and Matrix 2 genes to identify mutations that confer resistance to antiviral agents, including oseltamivir, zanamivir, and amantadine. Drug resistance mutations are listed in Supplementary Table S1.

### Barcode demultiplexing

The continuous improved data yield from each ONT flow cell permits users to make more efficient use and reduce cost by multiplexing several samples in a single sequencing run. Our previous work has shown that barcode contamination reads could account for <1% of the total reads from a multiplexed sequencing run (30), highlighting the need for careful barcode demultiplexing and the trade-off between sensitivity and specificity that applies to the demultiplexing methods. NanoSPC enables three demultiplexing modes, namely default-porechop, strict-porechop, and qcat. The default-porechop mode requires a barcode sequence to be present at either end of each read using porechop (https://github.com/rrwick/Porechop), thus maximizing the number of classified reads for downstream analysis. The strict-porechop mode requires the same barcode sequence to be present at both ends of each read, minimizing the number of misclassified reads. The qcat mode employs the demultiplexer offered by ONT and works similar to the default-porechop mode, due to the fact that porechop has been deprecated.

## IMPLEMENTATION

### Scalable and elastic computing

In order to perform reproducible and portable analysis of sequencing data, we implement NanoSPC using Nextflow pipeline manager (31) that enables parallel processing of multiple datasets (Figure 1). Moreover, all the software dependencies of the pipeline are packed within Docker images that can be executed on a wide variety of computing infrastructures.

Scalable Pathogon Pipeline Platform (SP^3^) is an open source web-based platform that we developed to host container-centric bioinformatic pipelines (32). We deploy NanoSPC to the SP^3^ platform to allow the pipeline to be run at high-performance computing (HPC) clusters and major commercial cloud platforms, such as Google Cloud, Amazon Elastic Computing Cloud (Amazon EC2), Microsoft Azure and OpenStack Cloud. SP^3^ has a cloud operation layer that provides interface to cloud platforms. A cloud agnostic scaler and scheduler allocate compute nodes based on CPU and memory requirement of the submitted jobs, and deallocate computational resources after the completion of the jobs. All SP^3^ software are deployed to a head node equipped with Ubuntu 18.04 and Nextflow. Compute nodes are only being built and used when tasks are sent to the head node. SP^3^ platform manages pipelines via a series of Web APIs, providing a range of functionalities, such as fetching data from European Nucleotide Archive (ENA) and other data sources, and excuating data analysis.

### Web interface and stand-alone application

We build a web interface for users to interact with cloud platforms via SP^3^ and perform data analysis (Figure 1). An authorised user can login to the SP^3^ system to upload their Nanopore sequencing data and execute the pipeline. Users can monitor the progress of the submitted job in real-time. The interface displays detailed logs, running commands, output files being generated, processing time, CPU usage, and memory usage for each individual process in the pipeline. Upon job completion, a complete run report displays the analysis results for each dataset. Users can choose to download the result files in bulk or only files that are of interest through running a command provided in the interface. The interface is served by the Nginx web server, configured to authenticate against web APIs, allowing user access control via web LDAP authentication. The web interface is written in python.

For users wishing to use NanoSPC to analyze data locally, we build Docker images that wrap the entire pipeline into a single environment. Users can download the Docker images and run command lines to perform the analysis.

### Availability

Details of web interface for cloud-based analysis and stand-alone application are available at https://nanospc.mmmoxford.uk.

### Usage

**Figure 2.**
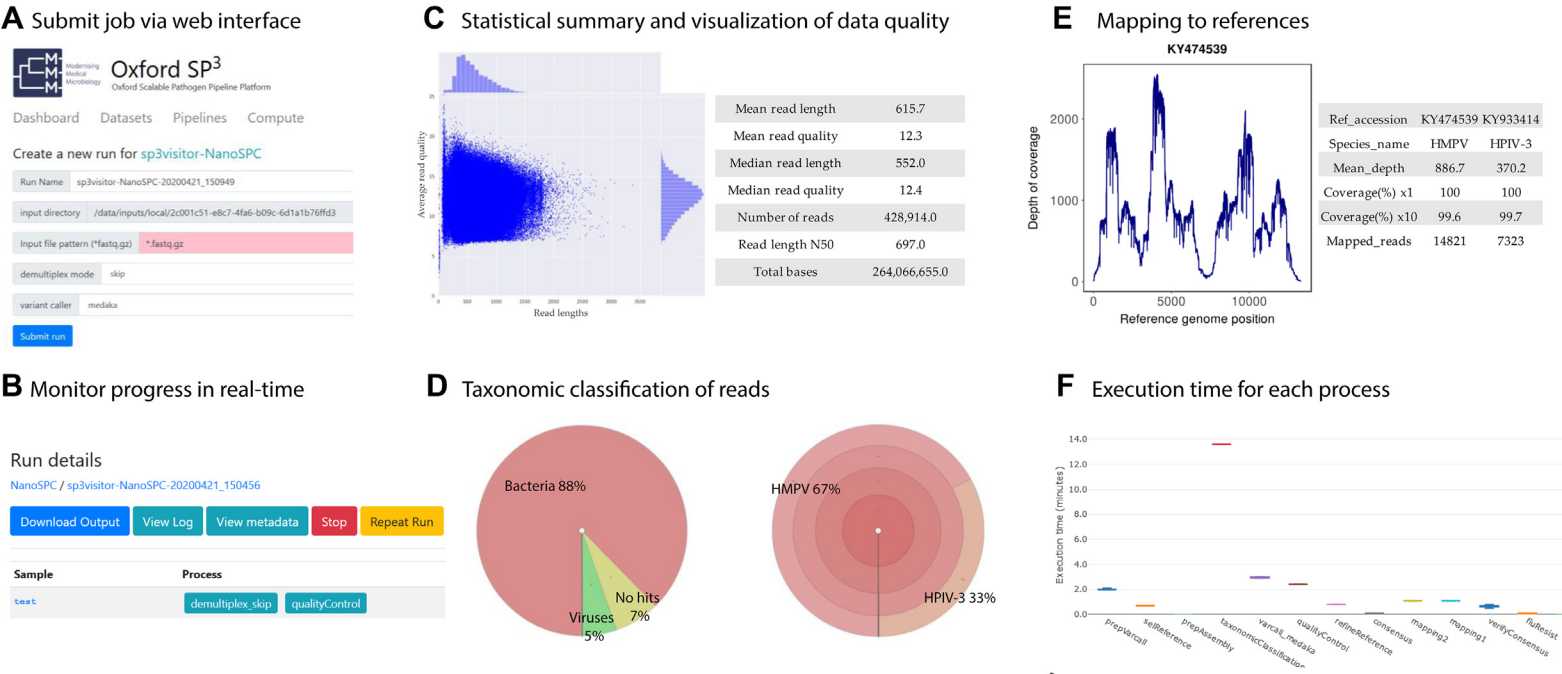
Example showing cloud-based analysis of Nanopore metagenomic sequencing data via NanoSPC. (**A**) and (**B**) Web interfaces for executing data analysis and real-time monitoring of the progress. (**C**) Statistical summary of the data quality. (**D**) Taxonomic assignment of sequencing reads, percentage of bacterial and viral reads. (**E**) Genome coverage by mapping sequencing reads to reference sequences. (**F**) Execution time for each process in the pipeline.

We illustrate the usage of NanoSPC with an exemplar Nanopore metagenomic sequencing dataset. The dataset contains Nanopore metagenomic sequencing reads generated from a clinical respiratory sample. The sample has been tested positive for human metapneumovirus (hmpv) in the clinical diagnostic laboratory as described in (26). Web interfaces for submitting the jobs to cloud-based analysis and real-time monitoring of the progress are shown in Figure 2A and B. Analysis of the data shows that Nanopore sequencing generated 428 914 reads with mean length of 615 bp (Figure 2C). Taxonomic classification of the sequencing reads are displayed using the Krona plots (Figure 2D). In this case, bacterial and viral reads accounted for 88% and 5% of the total sequencing reads. Among the viral reads, 67% and 33% are identified as hmpv and human parainfluenza virus type 3 (hpiv-3) reads, exemplifying simultaneous identification of multiple potentially pathogenic species from individual metagenomic sequencing dataset. Mapping to reference sequences showed that 14 821 hmpv reads cover the complete genome at mean coverage of 886, and 7323 hpiv-3 reads cover the complete genome at mean coverage of 370 (Figure 2E). The medaka mode is employed for calling variants, the total execution time is 13 mins (Figure 2F).

## CONCLUSION

We report NanoSPC, a scalable, portable, and cloud compatibilable pipeline for analyzing metagenomic sequencing data generated using ONT. NanoSPC differs from other pipelines, such as NanoPipe (33), by analyzing metagenomic sequencing data and identifying a range of organisms without a priori knowledge of species contained in the data. The implementation of cloud computing enables NanoSPC to increase the ease and efficiency of analysis of high throughput Nanopore metagenomic data. The analysis results can potentially be used in multiple clinical and public health applications.

We have applied NanoSPC to identify and assemble genomes of a range of pathogen species, including influenza viruses and human metapneumovirus, from Nanopore metagenomic sequencing data of clinical samples (29). The generated sequences have been used to investigate drug resistance and genetic diversity of influenza viruses from a UK hospital during the 2018/19 influenza season. The sequences also provided high resolution characterization of nosocomial transmission of influenza A virus and human metapneumovirus, contributing to improvement of infection prevention and control practices (26).

Our future step is to include prediction of antiviral drug resistance for viral species, such as influenza A virus, and phylogenetic analysis in the pipeline. Other features including discovery of novel pathogen species, particularly viruses, are also under investigation. ONT is undergoing constant improvement in sequencing chemistry and library preparation methods. ONT has released a new sequencing chemistry (R10 version flow cell) that provides improved accuracy. Our pipeline should be able to process R10 data but validating tests have not been conducted due to data availability. We are committed to continuously update our pipeline with the development of bioinformatics tools to enhance the accuracy and efficiency of the data analysis.

## Supplementary Material

gkaa413_Supplemental_File
